# Mendelian Randomisation: Concepts, Opportunities, Challenges, and Future Directions

**DOI:** 10.5334/gh.1438

**Published:** 2025-06-17

**Authors:** Sophie C. de Ruiter, Lena Tschiderer, Diederick E. Grobbee, Peter Willeit, Hester M. den Ruijter, A. Floriaan Schmidt, Sanne A. E. Peters

**Affiliations:** 1 Julius Center for Health Sciences and Primary Care, University Medical Center Utrecht, Utrecht University, Utrecht, The Netherlands; 2 Institute of Clinical Epidemiology, Public Health, Health Economics, Medical Statistics and Informatics, Medical University of Innsbruck, Innsbruck, Austria; 3 Ignaz Semmelweis Institute, Interuniversity Institute for Infection Research, Medical University of Vienna, Vienna, Austria; 4 Department of Public Health and Primary Care, University of Cambridge, Cambridge, United Kingdom; 5 Laboratory of Experimental Cardiology, Department of Cardiology, University Medical Center Utrecht, University Utrecht, Utrecht, The Netherlands; 6 Department of Cardiology, Amsterdam Cardiovascular Sciences, Amsterdam University Medical Centres, University of Amsterdam, Amsterdam, The Netherlands; 7 Institute of Cardiovascular Science, Faculty of Population Health, University College London, London, United Kingdom; 8 Division Heart and Lungs, Department of Cardiology, University Medical Center Utrecht, Utrecht University, Utrecht, The Netherlands; 9 UCL British Heart Foundation Research Accelerator, London, United Kingdom; 10 The George Institute for Global Health, School of Public Health, Imperial College London, London, United Kingdom

**Keywords:** Mendelian randomisation, opportunities, challenges, perspective, subgroups, sex-specific, omics

## Abstract

Mendelian randomisation is an approach in genetic epidemiology that uses genetic variants as instrumental variables to investigate the causal relationship between genetically proxied exposures and health outcomes. During the last years, the number of published Mendelian randomisation studies increased tremendously. There are several opportunities of Mendelian randomisation including obtaining potential causal relationships between both exogenous and endogenous exposures and outcomes and for identifying and prioritising drug-targets to inform clinical trials. However, it is also important to be aware of its challenges. This includes the reliability of results under the assumptions on instrumental variables, being aware of potential biases, the correct and critical interpretation of findings and comparison to the results of randomised controlled trials, as well as the availability of genetic data on specific subgroups. This review provides a comprehensive overview of the opportunities and challenges of Mendelian randomisation and presents key future perspectives.

## Introduction

### The concept of causality

Understanding causal relationships helps to unravel disease etiology, which provides mechanistic understanding to inform drug development and preventative action, thereby guiding health policy and clinical practice. In 1986, Holland stated, ‘no causation without manipulation’ (1), emphasizing that manipulation of an exposure is essential to investigate the causal effect of an exposure on an outcome. However, many epidemiological studies are observational by design. Consequently, when assessing relationships between exposures and outcomes in observational studies, we cannot draw any conclusions about causality. One of the main reasons is that observational studies are prone to confounding, that is, there may exist third factors that are related to both the exposure and outcome of interest, which bias observational associations. Potential solutions are to adjust, restrict, or stratify statistical models. However, in reality, residual confounding may still exist after applying these methods, since either we do not know all confounding factors or we have not assessed them in our study. Accordingly, randomised controlled trials (RCTs) are the gold standard for assessing causality. The random allocation of participants to an intervention or control group should—with large enough sample sizes—ensure an equal distribution of known and unknown confounding factors. As such, the only difference between the groups is the exposure to the intervention. However, RCTs can be infeasible since they are often very expensive and time consuming and sometimes it may not be ethically justifiable to conduct them. Consequently, alternative methods are needed.

### Mendelian randomisation

**Figure 1:**
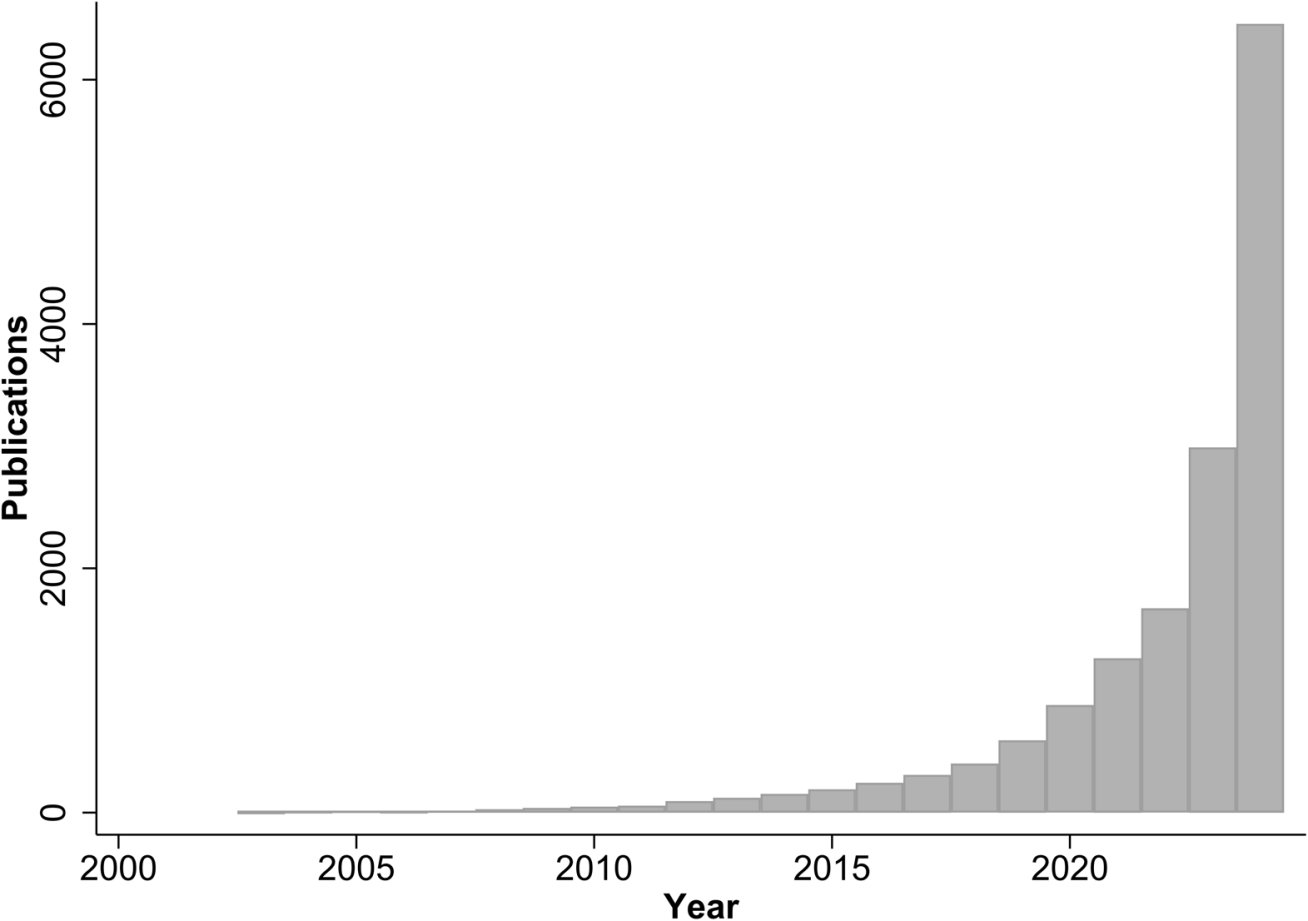
**Number of publications on Mendelian randomisation studies until 2024**. The data depicted in this figure have been extracted from PubMed on February 24^th^ 2025 using the search terms “Mendelian randomization” OR “Mendelian randomisation”. Publications from 2025 have been excluded.

Mendelian randomisation (MR), a specific application of instrumental variable analysis, lies in its ability to assess causality based on genetic data. The use of instrumental variables was introduced in 1928 in the context of econometrics (2). Building on these principles, the concept of MR was first described in 1986 (3), and MR was formally introduced in 2003 (4). The number of reported MR studies in scientific literature has grown exponentially every year since then. By February 2025, more than 15,000 articles using or describing MR had been published in PubMed (see Figure 1).

MR allows estimating the effect of an exposure on an outcome by selecting genetic variants (e.g., from GWAS data) strongly associated with the exposure of interest. These genetic variants serve as instrumental variables to proxy the exposure. Specifically, the effect size of the association of the genetic instrumental variable with the exposure and outcome, respectively, are used to estimate the causal effect of an exposure on an outcome (5,6). Since genetic variants are randomly allocated at gamete formation, MR is robust to confounding and reverse causation under three core assumptions: (1) the genetic variants are strongly associated with the exposure of interest (the relevance assumption); (2) there is no common cause between genetic variants and the outcome (the independence assumption); and (3) the genetic variants influence the outcome only through their effect on the exposure (i.e., absence of horizontal pleiotropy/exclusion restriction assumption).

### Interpretation of Mendelian randomisation study results

The main objective of many MR studies is to understand whether the relationship between an exposure and an outcome is causal or not. However, MR studies usually provide an effect size to estimate the causal effect of an exposure on an outcome (7). Its interpretation depends on the types of variables studied. In case the outcome of interest is binary, the effect size is often presented as odds ratio, while for continuous outcomes, beta coefficients are usually reported. If the exposure of interest is continuous, the relationship is often described as per unit or standard deviation increase or decrease and the exposure is referred to as “genetically proxied” or “genetically predicted”. For instance, a MR study investigating the link between interleukin 6 and coronary artery disease (8) found an odds ratio of 0.64 (95% confidence interval 0.54 to 0.76). The authors described their findings as ‘[g]enetically predicted circulating IL-6 levels were significantly inversely associated with CAD’ (8). In case the exposure of interest is categorical, the relationship can usually be interpreted per unit increase in the log odds of the exposure and the wording “genetic liability to” is often used. For instance, a MR study investigated the relationship between insomnia and different CVD outcomes and described their findings as “genetic liability to insomnia was associated with higher odds of six CVDs” (9).

Conducting MR analyses has become more accessible over the past years due to the availability of statistical tools, advanced software, and publicly accessible genetic data. This accessibility has allowed researchers to study both hypothesised and novel causal effects. For example, hypothesis-generating methods such as phenome-wide MR analyses systematically assess causality between a wide range of traits (10,11). This could uncover unexpected causal effects as a valuable starting point for further research. However, the relative ease of applying MR also comes with potential concerns. There is a fine line between meaningful rigorous exploration and what might be considered a phishing expedition, where the risk of chance findings or biased findings increases. Indeed, MR findings may be spurious or biased, and results are not always clinically relevant. An example of this is the relationship between schizophrenia and snoring. Using sex-specific GWAS data from up to 76,755 individuals with schizophrenia and 243,649 control individuals (12), as well as data from 408,317 UK Biobank participants including around 152,000 participants who reported snoring during sleep (13), we found that schizophrenia appears to be causally related to a lower risk of snoring in males (odds ratio 0.97, 95% confidence interval 0.95 to 0.99). Relationships like these are unlikely to have a direct preventive or clinical interpretation, as intervening is complex for several reasons, and such findings may reflect a bias rather than true causality. This underscores the importance of interpreting MR results cautiously, considering their clinical context and validity.

In the following sections, we will explain the opportunities of MR, elaborate on some of its challenges, and discuss future perspectives on its application. An overview is provided in Table 1.

**Table 1:** Opportunities, challenges, and future directions of Mendelian randomisation.


MENDELIAN RANDOMISATION

**Opportunities**

Causal relationships between exposures and outcomes

Drug-target prioritisation

**Challenges**

Validity of the assumptions

Selection bias

Comparison to randomised controlled trials

Mendelian randomisation within subgroups

**Future perspectives**

Integration of omics data

Availability of subgroup-specific genetic data

A subgroup-specific approach in multi-omics data integration


## Opportunities of Mendelian Randomisation

### Exposure-outcome relationships

Exposures assessed in MR can be both exogenous (i.e., originating outside the body such as environmental or lifestyle factors) and endogenous (i.e., originating within the body such as metabolic by-products or circulating proteins levels). Furthermore, one can study continuous exposures (e.g., levels of C-reactive protein [CRP]) as well as binary exposures (e.g., smoking behaviour). This provides a wide range of potential applications of MR.

MR can be used to study the causal effect of health-related behaviours (such as smoking and alcohol consumption) or complex biological traits (such as diabetes and obesity) on the risk of disease. For example, MR studies have shown that genetic liability to smoking initiation is related to a range of circulatory system diseases including aortic aneurysm, atrial fibrillation, coronary artery disease, and peripheral artery disease, among others, as well as to several digestive and nervous system diseases and cancer types (14). Additionally, higher genetically proxied body mass index has been linked to an increased risk for several cancer types including digestive system cancers, lung cancer, and uterine cancer (15). MR can also assess whether various types of circulating lipids, metabolites, or proteins are causally related to health outcomes. For example, CRP is observationally associated with coronary heart disease (CHD) (16), but prior to robust MR studies it was unknown whether this association was causal (17). MR analyses have shown that CRP is not likely to be a causal risk factor for CHD but rather a marker of the inflammation contributing to CHD (18,19). Similarly, in 1989, the causal relationship between fibrinogen and myocardial infarction was studied by using a polymorphism in the fibrinogen beta-gene as a proxy for plasma fibrinogen levels (20), implicitly applying the principles of what was later formally introduced as MR. The study found that increased fibrinogen levels associated with the genetic factor did not increase the risk of myocardial infarction. In addition to circulating constituents of the blood, interest has also emerged in other biomarkers as exposure of interest, which can provide additional insights into biological changes associated with disease processes. For example, metabolites measured in urine could serve as non-invasive markers of cardiovascular disease (CVD), which may be used in prognosis or diagnosis. Messenger ribonucleic acid (mRNA) expression is another example of a potential exposure of interest in MR and provides deeper insights into the causal pathways linking genes to disease. GWASs have identified thousands of genetic variants associated with complex diseases, including CVD. By using mRNA as an exposure in MR studies, it becomes possible to identify genes with expression causally related to disease (21). Using tissue-level mRNA expression data as an exposure in MR allows for a further refined understanding of the biological mechanisms underlying GWAS-identified associations (22,23,24,25,26). For example, in the context of chronic kidney disease, recent analyses of renal transcriptomes have demonstrated the causal effects of kidney-specific gene expression on kidney function traits (25).

### Drug-target prioritisation

MR can also be used for drug-target prioritisation. When a potential drug-target is the exposure of interest, MR is referred to as drug-target MR. While traditional genome-wide MR selects instrumental variables from genetic variants across the genome, drug-target MR selects instrumental variables from genetic variants in or around the gene encoding the drug-target (27). This approach can identify novel drug-targets and prioritise proteins for further validation in registry data studies, wet lab studies, and eventually, RCTs. Previous research has shown that genetically supported targets have higher success rates in phases II and III clinical trials (28,29), and given the size of the human proteome, prioritisation (and deprioritisation) of potential targets can streamline drug development, optimizing resource allocation and reducing the risk of costly late-stage trial failures (30,31). In addition to (de)prioritisation, drug repurposing—defined as identifying new therapeutic uses for existing and approved drugs—has gained attention (32).

## Challenges in Mendelian Randomisation

Despite the opportunities of MR to estimate causal effects of various types of exposures and the robustness of MR to most types of confounding, which affect observational study designs, MR also has important challenges and limitations.

### Validity of the MR assumptions

MR has three core assumptions, as described above. The relevance assumptions states that the instrumental variable are strongly related to the exposure of interest. In MR studies, an option to select genetic variants to be included in the instrumental variables is biological knowledge. For instance, it is possible to use variants within the *CRP* gene region to genetically proxy CRP levels (18). Alternatively, instrumental variables can be built based on results from large-scale GWASs. This has, for example, been done in a phenome-wide study on body mass index (33). When relying on GWAS findings, it is important that large-scale studies are available in order to construct valid instrumental variables. The second assumption of MR, the independence assumption, usually holds given that genotypes are defined at gamete formation and are rarely changed by environmental factors. However, there are factors that could invalidate this assumption including population structure or assortative mating, which are in general greater concerns for traits linked to social patterns (34). Another potential threat to the validity of the MR assumptions is horizontal pleiotropy, where selected genetic variants influence the outcome through pathways independently of the exposure of interest (35,36,37,38). This would violate the so-called exclusion restriction assumption. Moreover, genetic variants in linkage disequilibrium could be correlated with other variants related to a confounder that may affect the outcome (34). In general, it is not possible to fully exclude horizontal pleiotropy in an MR. However, there are methods that allow to further investigate potential horizontal pleiotropy such as MR-Egger (39) and MR-PRESSO (36).

### Selection bias

**Figure 2:**
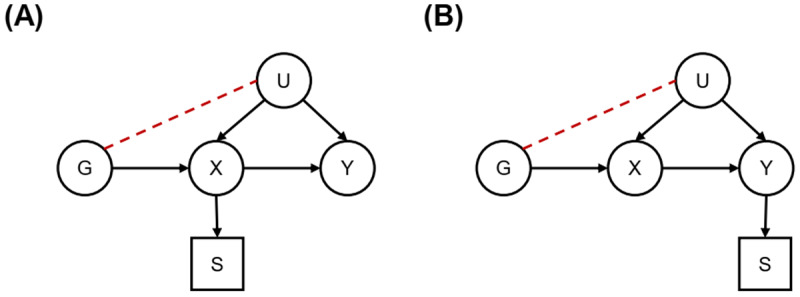
**Directed acyclic graphs on selection bias in Mendelian randomisation analyses**. Directed acyclic graphs where G represents the instrumental variable, X the exposure, U the confounder, Y the outcome and where selection (S) leads to selection bias if it is dependent on levels of the exposure (panel **A**) or outcome (panel **B**).

While the independence assumption usually holds in MR, the independence of the instrumental variable and potential confounders can be at risk. For example, conditioning on a variable that is influenced by both the instrumental variable and one or more of the confounders of the exposure-outcome association (i.e., conditioning on a collider) induces an association between the two (40), known as collider bias (Figure 2). If the exposure or the outcome of interest in MR is associated with increased mortality, selection of participants for MR is inherently dependent on the levels of the exposure or outcome, inducing selection bias—a form of collider bias. As such, selection bias in MR can lead to an association between the instrumental variable and the outcome in the absence of a causal effect of the exposure on the outcome (41). Methods have been developed to address this issue, but it cannot be ruled out completely (42).

### Mendelian randomisation versus randomised controlled trials

MR studies are often compared to RCTs. While both methods share a conceptual basis in randomisation (either to study arms in RCTs, or to genotypes in MR), they differ in several ways (43). One difference is the timing of randomisation. In RCTs, participants are randomly allocated to a study arm soon after study recruitment, ensuring that well-conducted RCTs are free of confounding and selection bias. MR, in contrast, relies on the random allocation of genetic material at gamete formation, which occurs typically long before study recruitment. Consequently, MR can also be affected by selection bias, specifically survivor bias, as participants need to be survivors of their genotype. However, this is of course also the case for being included in an RCT. Furthermore, individuals with high-risk traits may have received interventions that may modify their phenotypic trait or their disease risk later in life. Consequently, this needs to be taken into account when performing GWAS as well as MR studies. For instance, a large-scale GWAS for blood pressure traits adjusted their analyses for antihypertensive medication use (44). Another consequence of the difference in timing of randomisation is that in MR, genetic variants used to proxy the exposure of interest may have a lifelong cumulative effect, while a RCT estimates the effect of an intervention administered over a specific and usually shorter time frame (43). This implies that MR estimates cannot be compared directly with effect sizes from RCTs. Furthermore, this MR may not be valid to assess disease risk due to acute alterations of risk factors (43).

As mentioned above, MR can also be applied to drug prioritisation. However, there are several challenges of drug-target MR (5,45). A crucial initial step in drug-target MR is the selection of the underlying instrumental variable, which can be based on protein quantitative trait loci (pQTL), expression quantitative trait loci (eQTL) data, or on biomarker data. For instance, if one wants to apply drug-target MR to inform on potential effects of proprotein convertase subtilisin/kexin type 9 (PCSK9) inhibitors, one could study variations in the *PCSK9* gene by either using eQTL data on PCSK9 expression, pQTL data on the PCSK9 protein, or genetic variants related to the underlying biomarker (i.e., low-density lipoprotein cholesterol) within the *PCSK9* gene region. Each of these scenarios has its advantages and drawbacks (5,45). In general, it is important to note that drug-target MR may help to inform clinical trials but cannot replace them (43). For instance, drug-target MR relies on the principle that the underlying drugs work via a specific target. However, many drugs have so-called pleiotropic or off-targets effects, which may be missed when only studying genetic variants in a specific gene region. For these reasons, MR and RCTs are complementary and not interchangeable and validation is required before MR results can be translated to clinical practice. Ultimately, RCTs may follow, which will provide additional insights in whether targeting the identified drug-targets can reverse the disease process or prevent disease onset.

### Subgroup-specific GWAS data

Another limitation to the application of MR is the limited availability of subgroup-specific GWAS data, such as sex-specific or ancestry-specific data, which are essential for conducting subgroup-specific MR. Sex-specific MR is relevant because sex-combined MR can mask potential sex differences in either the direction or magnitude of causal effects, resulting in estimates that may not hold true for either of the sexes (46). For example, when using MR to estimate causal risk factor effects, a sex-specific approach may be valuable at a population level to know if targeting risk factors in a sex-specific way is needed to obtain similar levels of risk reduction across the full population. Similarly, when performing MR to estimate the effects of protein perturbation, sex-stratification is relevant because protein expression levels, protein function and the effect of protein perturbation may differ between females and males.

Sex-specific MRs are best performed using sex-specific GWAS, as heterogeneity in genetic effects between sexes may invalidate MR analysis relying on sex-combined GWAS estimates for the association of the instrumental variables with the exposure of interest. Notably, recent UK Biobank analyses found that 70% of non-binary and almost 10% of binary traits had at least one autosomal genetic variant showing a significantly different association in women than in men (at a *p* < 1 × 10^–8^) (47). Sex-differentiated genetic effects were confirmed, for example, for schizophrenia (48), serum uric acid concentrations (49), waist-hip ratio (50), and central obesity and adipose distribution (51). Although the first sex-stratified GWAS was reported in 2008 (reporting genetic associations with height), sex-stratification in GWAS is not common practice. Even in the GWAS that do stratify on sex, challenges may persist. For example, coronary artery disease GWAS in females often suffer from weaker statistical power as compared to the GWAS in males, due to differences in disease occurrence (52) as well as smaller effect sizes. Although the majority of coronary artery disease is caused by atherosclerosis, pathophysiological differences in coronary artery disease between sexes such as spontaneous coronary artery dissection (SCAD) in women may be the reason for smaller effect sizes as the genetic architecture of SCAD is distinct (53). In addition, GWAS may suffer from misclassification of cases, as some coronary artery disease subtypes such as ischemia with no obstructive arteries (INOCA) may not be captured under standard outcome definitions misclassifying patients with INOCA under the “control” group (54).

## Future Perspectives

### Availability and integration of omics data

A promising future opportunity of MR is the integration of ‘omics data. In the future, technological advancements are likely to increase the scale and scope of omics studies such as proteomics, transcriptomics, and metabolomics. Despite the important role of proteins in disease pathogenesis, proteomic studies at a large scale have only recently become feasible (55,56,57,58,59,60). These pQTL studies have enabled research into the causal effect of proteins on disease. Potential remains in scaling up these studies in terms of both sample size and coverage of the proteome. Moreover, blood is the primary source of current pQTL studies. While blood is most accessible, it may not reflect protein levels in tissues more directly involved in disease processes, highlighting the need for more tissue-specific studies. In addition to proteomics, other types of omics data provide further insights and expand the applications of MR. As described above, transcriptomics (RNA-sequencing) can be used within MR to study the effects of gene expression, and metabolomics can be used to further improve understanding of metabolic changes involved in disease progression. Additionally, methylation data allows to study regulatory mechanisms of gene expression (61). Such omics studies, with expected advancements in sample size, coverage, and quality, are expected to provide novel opportunities for MR to study disease mechanisms and identify therapeutic targets.

Future MR efforts to unravel disease mechanisms and identify novel drug targets or repurposing opportunities should focus on triangulating evidence from various types of omics data, using publicly available data supported by open science practices (62). The integration of multi-omics data provides a comprehensive understanding of complex biological systems, as each type of omics data offers different insights and poses different assumptions regarding for example pleiotropy within MR (5). In line with the notion of triangulation in epidemiology where multiple lines of evidence are needed to support robust conclusions, combining multiple type of omics within MR can reduce the limitations of individual approaches and achieve a more complete view of the disease mechanisms.

### Availability of subgroup-specific GWAS data

The field of MR is rapidly evolving, and both sex-stratified and other subgroup-stratified MR studies are likely to become more common when advancements in genotyping and growing computational power drive the availability of subgroup-specific GWAS data. Currently, sex stratification in GWAS is not the common practice, probably because it is not considered to be a priority or because of concerns about a loss of statistical power from smaller sample sizes. However, combining results of sex-specific GWAS in a meta-analysis has shown to be effective in overcoming this potential loss of power (46). Large consortia, such as DIAGRAM (63) and MAGIC (64), have already published such meta-analyses of sex-specific GWAS data for various glycaemic traits and diabetes, enabling to study sex-specific effects and test for sex differences in effects that might otherwise be obscured in combined analyses. The same applies to ancestry-specific GWAS data. Although many GWAS are still conducted in European populations, there is growing recognition that performing GWAS across multiple ancestries is essential because of genetic heterogeneity across ancestries (65,66) and ancestry-specific GWAS studies have already been published (67,68,69,70).

### A subgroup-specific approach in multi-omics data integration

Combining the two research fields of subgroup-specific and multi-omics approaches would be specifically valuable. For instance, previous studies have shown sex differences in gene expression across various human tissues (71,72), and found that sex influences biological gene networks through variations in gene co-expression (73). These findings suggest that sex-specific biological mechanisms may influence the efficacy of existing treatments. Novel therapeutic strategies, informed by sex-specific multi-omics analyses, may better target underlying pathways when they are unique to females or males or different between the sexes, offering more effective prevention and treatment options. However, such sex-specific analyses rely on sex-specific data, which are often not available. For example, numerous pQTL for plasma proteins have been identified, but studies reporting pQTL in a sex-stratified population remain scarce. To bridge this gap, we emphasize the need for sex-specific data, not only for risk factors, but also for proteomics, metabolomics, transcriptomics, and other omics data.

## Conclusions

The framework of MR, a type of instrumental variable analysis, allows estimating relationships between exposures and outcomes based on genetic variants. It has a wide range of application, including the identification of potential causal associations between risk factors and diseases and the prioritisation of drug-targets. However, with the growing number of MR studies published, it is important to acknowledge its challenges. Large-scale subgroup-specific GWAS and multi-omics approaches are still needed to better understand specific disease mechanisms. This could increase our ability to address the complexities of diseases and guide the development of more personalised therapeutic strategies, ultimately improving individual health.
